# The impact of enriched environments on cerebral oxidative balance in rodents: a systematic review of environmental variability effects

**DOI:** 10.3389/fnins.2024.1366747

**Published:** 2024-04-11

**Authors:** Tiago Lacerda Ramos, Matheus Santos de Sousa Fernandes, Débora Eduarda da Silva Fidélis, Gabriela Carvalho Jurema Santos, Renata B. Albuquerque, Diorginis José Soares Ferreira, Raphael Fabrício de Souza, Georgian Badicu, Fatma Hilal Yagin, Burak Yagin, Reem M. Alwhaibi, Fabrício Oliveira Souto, Claúdia Jacques Lagranha

**Affiliations:** ^1^Programa de Pós-Graduação em Biologia Aplicada à Saúde, Centro de Biociências, Universidade Federal de Pernambuco, Recife, Pernambuco, Brazil; ^2^Instituto Keizo Asami, Universidade Federal de Pernambuco, Recife, Pernambuco, Brazil; ^3^Programa de Pós-Graduação em Nutrição, Universidade Federal de Pernambuco, Recife, Pernambuco, Brazil; ^4^Physical Education Department/Federal University of São Francisco Valley, Petrolina, Pernambuco, Brazil; ^5^Department of Physical Education, Federal University of Sergipe, São Cristovão, Sergipe, Brazil; ^6^Department of Physical Education and Special Motricity, Transilvania University of Braşov, Braşov, Romania; ^7^Department of Biostatistics and Medical Informatics, Faculty of Medicine, Inonu University, Malatya, Türkiye; ^8^Department of Rehabilitation Sciences, College of Health and Rehabilitation Sciences, Princess Nourah Bint Abdulrahman University, Riyadh, Saudi Arabia; ^9^Programa de Pós-Graduação em Nutrição Atividade Física e Plasticidade Fenotípica, Centro Acadêmico de Vitória, Vitória de Santo Antão, Pernambuco, Brazil

**Keywords:** enriched environment, oxidative stress, brain, central nervous system, biochemistry, antioxidants

## Abstract

**Introduction:**

The present review aimed to systematically summarize the impacts of environmental enrichment (EE) on cerebral oxidative balance in rodents exposed to normal and unfavorable environmental conditions.

**Methods:**

In this systematic review, four databases were used: PubMed (830 articles), Scopus (126 articles), Embase (127 articles), and Science Direct (794 articles). Eligibility criteria were applied based on the Population, Intervention, Comparison, Outcomes, and Study (PICOS) strategy to reduce the risk of bias. The searches were carried out by two independent researchers; in case of disagreement, a third participant was requested. After the selection and inclusion of articles, data related to sample characteristics and the EE protocol (time of exposure to EE, number of animals, and size of the environment) were extracted, as well as data related to brain tissues and biomarkers of oxidative balance, including carbonyls, malondialdehyde, nitrotyrosine, oxygen-reactive species, and glutathione (reduced/oxidized).

**Results:**

A total of 1,877 articles were found in the four databases, of which 16 studies were included in this systematic review. The results showed that different EE protocols were able to produce a global increase in antioxidant capacity, both enzymatic and non-enzymatic, which are the main factors for the neuroprotective effects in the central nervous system (CNS) subjected to unfavorable conditions. Furthermore, it was possible to notice a slowdown in neural dysfunction associated with oxidative damage, especially in the prefrontal structure in mice.

**Discussion:**

In conclusion, EE protocols were determined to be valid tools for improving oxidative balance in the CNS. The global decrease in oxidative stress biomarkers indicates refinement in reactive oxygen species detoxification, triggering an improvement in the antioxidant network.

## 1 Introduction

It is well known that vulnerability to oxidative damage varies among organs, with the brain being one of the most susceptible to oxidative stress (OS) (Halliwell, 2006). Characterized by the imbalance between the levels of pro-oxidant and antioxidant, OS is commonly related to the pathogenesis of several diseases, including Alzheimer’s, Parkinson’s, schizophrenia, and stroke (Chen and Zhong, 2014; Orellana-Urzúa et al., 2020; Ermakov et al., 2021; Balietti and Conti, 2022).

Seeking strategies to minimize OS and even combat pathologies-associated, non-pharmacological strategies have been suggested to attenuate cellular damage induced by OS in different organisms (Wronka et al., 2022; Sharma and Mehdi, 2023) by changing the lifestyle as well as the consumption of specific foods and vitamins (Gomes et al., 2017; Sharma and Mehdi, 2023). Thus, it is postulated that a rich environment can boost mental and physical health and, therefore, attenuate OS by reducing the production of pro-oxidative compounds, generally termed reactive oxygen species (ROS), while increasing their scavenger through antioxidant systems, assembled by enzymatic and non-enzymatic compounds (Fernandes et al., 2022).

The EE paradigm emerged in 1947 through Donald Hebb, who studied animal behavior and realized that the variability of the environment was related to neurological and behavioral improvements. The EE consists of an environment (cage) assembled by inanimate objects varying in shapes and textures, increased social interaction, higher voluntary physical activity, and continuous exposure to learning activities, enhancing both cognitive function and sensory motor aspects. Furthermore, EE upregulates processes linked to neuroplasticity such as neurogenesis, synaptogenesis, and neurotrophin production, culminating in a protective effect against neurodegeneration (Olson et al., 2006; Kempermann, 2019).

Although some mechanisms of EE intervention have been elucidated, there are still several gaps in the literature. Due to the variability of protocols in relation to the number of objects, number of animals, and cage dimensions (width, depth, and length), it is important to clarify the impacts of this variability on OS biomarkers and antioxidant defenses in rodent tissues. Therefore, this study aimed to systematically summarize the impacts of EE on cerebral oxidative balance in rodents.

## 2 Methods

The review followed the Preferred Reporting Items for Systematic Reviews and Meta-Analysis (PRISMA) guidelines.

### 2.1 Study selection and eligibility

Eligibility criteria were previously used to minimize the risk of bias. The inclusion and exclusion criteria followed the Population, Intervention, Comparison, Outcomes, and Study (PICOS) (Table 1). There were no restrictions on language or publication date. The following inclusion criteria were used: (a) rodent studies, (b) evaluation of oxidative balance parameters, (c) absence of a control group or comparator, and (d) Studies with any other animal model and biological organism were not used, reviews, letters to editors, duplicates and the presence of data used in different studies were excluded.

**Table 1:** PICOS strategy.

	Inclusion criteria	Exclusion criteria
Population	Rodents	Human and other organisms
Intervention	Environmental enrichment	Non-environmental enrichment
Control	Non-environmental enrichment	Any other comparison group
Outcomes	Carbonyls, 2′,7′-dichlorofluorescein, malondialdehyde, nitrotyrosine, reactive oxygen species (ROS levels), 4-hydroxynonenal, and superanion. antioxidant outcomes include catalase, ferric reducing antioxidant power, glutathione S-transferase, reduced glutathione, oxidized glutathione; reduced glutathione/oxidized glutathione ratio; glutathione peroxidase; copper/zinc superoxide dismutase, SOD-2 (MnSOD), total radical antioxidant	No oxidati contribution ve Balance parameters
Study design	Animal studies	Reviews; case reports; letters to the editor; comments, etc.

### 2.2 Information sources and search strategy

The search strategy was carried out during the period from April to May 2023. The databases used were PubMed (Medline), Scopus, and Embase. The search strategies used were PubMed (Medline): [(Environmental Enrichment) OR (Enriched Environment)] AND ((((((Oxidative Stress) OR (Stress, Oxidative)) OR (Oxidative Damage)) OR (Oxidative Damages)) OR (Oxidative Injury)) OR (Oxidative Injuries)). In the Embase, Scopus, and Science Direct databases, the following search equation was used: ((“Environmental Enrichment”) OR (“Enriched Environment”)) AND ((((((“Oxidative Stress”) OR (“Stress, Oxidative”)) OR (“Oxidative Damage”)) OR (“Oxidative Damages”)) OR (“Oxidative Injury”)) OR (“Oxidative Injuries”)).

### 2.3 Selection and data collection process

The screening of studies was performed by reading the title, abstract, and full text. The selection of studies was performed by two independent researchers (MSSF and TLR). The discrepancies were resolved by a third rater. Data were extracted by two independent researchers. The discrepancies were resolved by a third author (FOS).

### 2.4 Items

To answer the hypothesis of this systematic review, different data were extracted. Initially, we collected the following information: author, year, species, sex, and age. In addition, data were collected on the structure of the environmental enrichment (EE) protocol, including the number of animals per cage, housing dimensions (length, width, and depth or height), and the time of exposure to the EE. Next, data on brain tissues and OS biomarkers were evaluated, such as carbonyls, 2′,7′-dichlorofluorescein (DCF), malondialdehyde (MDA/TBARS), nitrotyrosine, ROS levels, 4-hydroxynonenal (4-HNE), and superanion. Antioxidant outcomes include catalase, ferric reducing antioxidant power (FRAP), glutathione S-transferase (GST), reduced glutathione (GSH), oxidized glutathione (GSSG), reduced glutathione (GSH)/oxidized glutathione (GSSG) ratio, glutathione peroxidase (GPx), copper/zinc superoxide dismutase (Cu/Zn SOD), superoxide dismutase (SOD), SOD-2 (MnSOD), and total radical antioxidant.

### 2.5 Methodological quality assessment

The SYRCLE’s strategy was used to assess the methodological quality of the animal studies. The tool consisted of 10 questions that evaluated methodological criteria: (Q1)—Was the allocation sequence adequately generated and applied? (Q2)—Were the groups similar at baseline or were they adjusted for confounders in the analysis? (Q3)—Was the allocation to the different groups adequately concealed? (Q4)—Were the animals randomly housed during the experiment? (Q5)—Were the caregivers and/or investigators blinded by the knowledge of which intervention each animal received during the experiment? (Q6)—Were animals selected at random for outcome assessment? (Q7)—Was the outcome assessor-blinded? (Q8)—Were incomplete outcome data adequately addressed? (Q9)—Are reports of the study free of selective outcome reporting? (Q10)—Was the study free of other problems that could result in a high risk of bias? Questions were answered with options of “Yes,” “No,” or “Not clear.” When the answer was “yes,” a score was given; when the answer was “no” or “not clear,” no score was given. The overall scores for each article were calculated as a score of 0–10 points, with the quality of each study being classified as high (8–10), moderate (5–7), or low (<5). The two authors independently reviewed all the included studies. Discrepancies between authors were resolved by consensus. The quality outcomes are described in Table 2.

**Table 2:** Methodological quality assessment.

Author, year	Q1	Q2	Q3	Q4	Q5	Q6	Q7	Q8	Q9	Q10
Cechetti et al. (2012)	Y	Y	Y	Y	N	Y	N	Y	Y	Y
Cheng et al. (2014)	Y	Y	Y	Y	N	Y	N	Y	Y	Y
Fernández et al. (2004)	Y	Y	Y	Y	N	Y	N	Y	Y	Y
Herring et al. (2010)	Y	U	Y	Y	N	Y	N	Y	Y	Y
Jain et al. (2012)	Y	Y	Y	Y	N	Y	N	Y	Y	Y
Prado Lima et al. (2018)	Y	Y	Y	Y	N	Y	N	Y	Y	Y
Mármol et al. (2015)	Y	Y	Y	Y	N	Y	N	Y	Y	Y
Mármol et al., 2017	Y	Y	Y	Y	N	Y	N	Y	Y	Y
Molina et al. (2021)	Y	Y	Y	Y	N	Y	N	Y	Y	Y
Montes et al. (2019)	Y	Y	Y	Y	N	Y	N	Y	Y	Y
Muhammad et al. (2017)	Y	U	Y	Y	N	Y	N	Y	Y	Y
Pereira et al. (2009)	Y	Y	Y	Y	N	Y	N	Y	Y	Y
Tapias et al. (2022)	Y	Y	Y	Y	N	Y	N	Y	Y	Y
Thamizhoviya and Vanisree (2021)	Y	U	Y	Y	N	Y	N	Y	Y	Y
Zhang et al. (2016)	Y	Y	Y	Y	N	Y	N	Y	Y	Y
Zhang et al. (2021)	Y	Y	Y	Y	N	Y	N	Y	Y	Y

Q1: Was the allocation sequence adequately generated and applied?; Q2: Were the groups similar at baseline or were they adjusted for confounders in the analysis?; Q3: Was the allocation to the different groups adequately concealed?; Q4: Were the animals randomly housed during the experiment?; Q5: Were the caregivers and/or investigators blinded from knowledge of which intervention each animal received during the experiment?; Q6: Were animals selected at random for outcome assessment?; Q7: Was the outcome assessor blinded?; Q8: Were incomplete outcome data adequately addressed?; Q9: Are reports of the study free of selective outcome reporting?; and Q10: Was the study apparently free of other problems that could result in a high risk of bias? Y, Yes; N, No; U, Unclear.

## 3 Results

### 3.1 Search results

**Figure 1:**
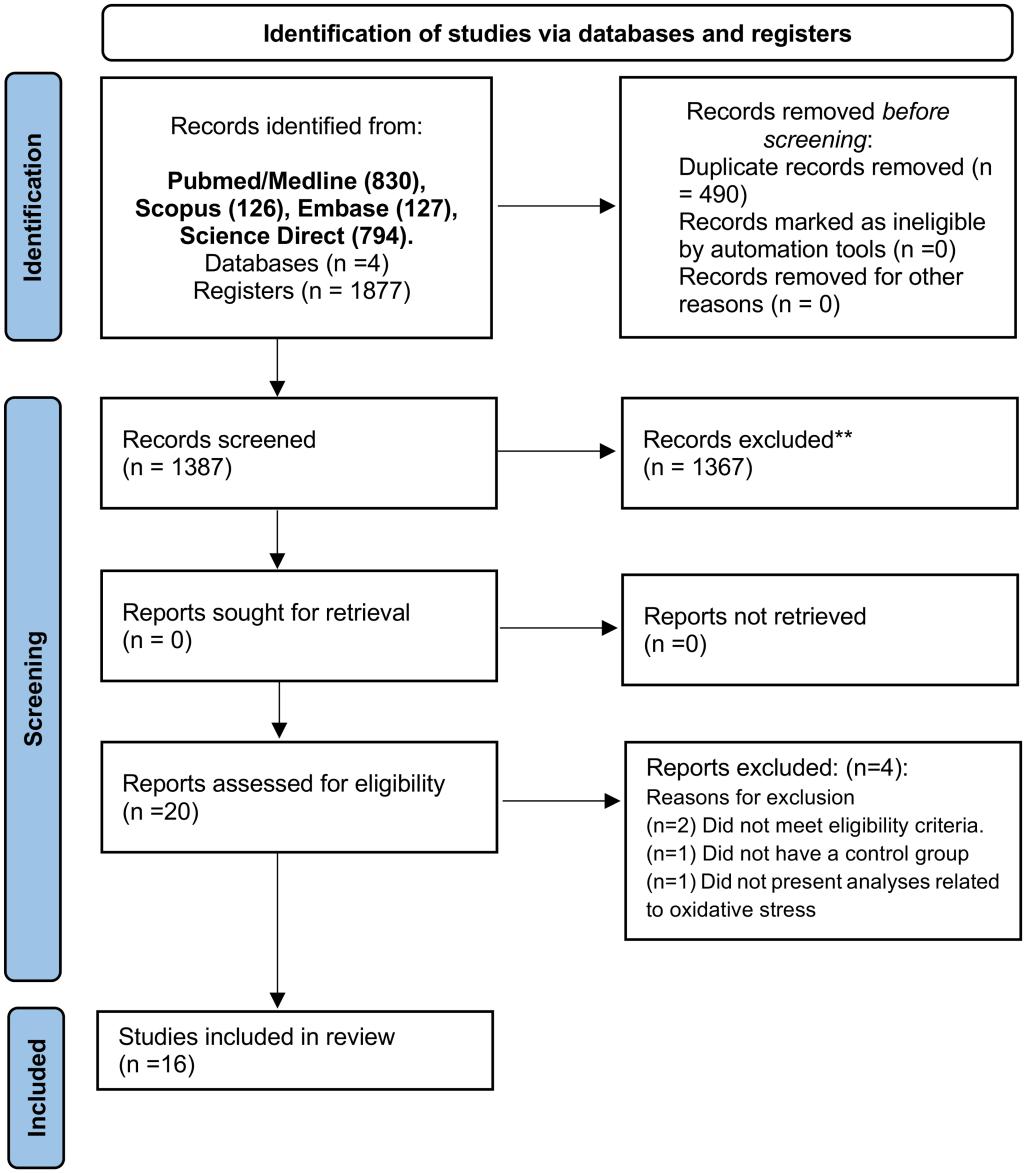
PRISMA flow diagram. ^*^Consider, if feasible to do so, reporting the number of records identified from each database or register searched (rather than the total number across all databases/registers). ^**^If automation tools were used, indicate how many records were excluded by a human and how many were excluded by automation tools. From Page et al. (2021). For more information, visit: http://www.prisma-statement.org/.

In an initial search, 1,877 articles were identified [PubMed/Medline (830), Scopus (126), Embase (127), and Science Direct (794)]. Then, 346 duplicates were excluded using the EndNote® software. Then, 424 articles were screened and submitted to the eligibility criteria, and 409 articles were excluded based on title and abstract reading. Twenty studies remained for full-text reading. Four studies were excluded due to the following reasons: Two did not agree with the eligibility criteria, one study did not have a control group, and one study did not perform specific analyses of OS. Finally, 16 studies were included in this systematic review (Figure 1).

### 3.2 Methodological quality assessment

The results of the methodological quality assessment of the included studies are shown in Table 2. All studies showed adequate and randomized allocation with randomly selected animals. In addition, incomplete results were handled appropriately, free from selective results and bias. As these are studies involving intervention (EE), it is not possible to consider the investigation and analysis of the results blindly. In general, all studies presented satisfactory quality criteria.

### 3.3 Study characteristics

The studies included in this systematic review were published between the years 2004 and 2022. Initially, we observed that the studies used different species of rodents (rats and mice). Five studies used Sprague Dawley rats (Fernández et al., 2004; Jain et al., 2012; Zhang et al., 2016, 2021; Tapias et al., 2022). Five studies used Wistar rats (Pereira et al., 2009; Cechetti et al., 2012; Prado Lima et al., 2018; Molina et al., 2021; Thamizhoviya and Vanisree, 2021). Two studies used Long-Evans rats (Mármol et al., 2015, 2017). Two studies used Swiss mice (Muhammad et al., 2017; Montes et al., 2019). One study used Kunming mice (Cheng et al., 2014), and one study used TgCRND8 mice (Herring et al., 2010). Regarding gender, 10 included studies used male individuals only (Pereira et al., 2009; Cechetti et al., 2012; Jain et al., 2012; Zhang et al., 2016, 2021; Mármol et al., 2017; Prado Lima et al., 2018; Montes et al., 2019; Thamizhoviya and Vanisree, 2021; Tapias et al., 2022). Five studies used both sexes (Fernández et al., 2004; Cheng et al., 2014; Mármol et al., 2015; Muhammad et al., 2017; Molina et al., 2021). Only one study used the female sex (Herring et al., 2010).

Next, we analyzed the different characteristics of the EE protocols. The number of animals per cage varied in the included studies from 5 to 20 rodents. There was heterogeneity of objects inserted into the cages of the rodents, including ramps, three floors, running wheels, several objects, tunnels, plastic colored toys, shelters, balls, soft materials, varied locomotive substrates, tubes, boxes, bells, a climbing ladder, chain, and a screen cover with a color block and running wheel. The size of the cages in the included studies varied in length, width, depth, or height, and was expressed in either centimeters or inches. Additionally, the time of exposure to EE varied across the included studies, ranging from 7 days to 20 weeks.

Different brain areas were observed in the included studies. Twelve studies were conducted on the hippocampus (Fernández et al., 2004; Pereira et al., 2009; Cechetti et al., 2012; Jain et al., 2012; Cheng et al., 2014; Mármol et al., 2015, 2017; Prado Lima et al., 2018; Montes et al., 2019; Molina et al., 2021; Zhang et al., 2021; Tapias et al., 2022). One study evaluated the medial-temporal lobe cortex (MTLC) (Cheng et al., 2014). One study evaluated the total cortex and striatum (Fernández et al., 2004). One study evaluated the cerebral hemisphere (Herring et al., 2010). One study evaluated the cortex (Mármol et al., 2015). Two studies evaluated the prefrontal cortex (Zhang et al., 2016; Montes et al., 2019). One study evaluated the total brain (Mármol et al., 2017). One study evaluated the frontal cortex (Pereira et al., 2009). One study evaluated the forebrain (Thamizhoviya and Vanisree, 2021). Of the selected studies, five did not expose the animals to adverse environmental conditions (Cheng et al., 2014; Mármol et al., 2015, 2017; Muhammad et al., 2017), while 11 carried out the exposure to stimulating changes in the results of oxidative balance. The following exposures were used: chronic cerebral hypoperfusion (Cechetti et al., 2012), Alzheimer-like model (Herring et al., 2010), hypobaric hypoxia (Jain et al., 2012), amyloid beta neurotoxicity (Prado Lima et al., 2018), noise (Molina et al., 2021), toluene (Montes et al., 2019), hypoxia-ischemia (Pereira et al., 2009), traumatic brain injury (Tapias et al., 2022), oxidative damage (Thamizhoviya and Vanisree, 2021), hypoxia (Zhang et al., 2016), and post-stroke condition (Zhang et al., 2021) (Table 3).

**Table 3:** Sample and environmental enrichment protocol description.

Author, Year	Species, sex, and age	Animals per cage	Environmental enrichment protocol and housing dimensions (Length, width, and depth or height)		Exposure time to environmental enrichment
	
Cechetti et al. (2012)	Wistar rats; Male; age group were not informed	8	Ramps; Three floors; Running Wheels and Several objects	40 cm × 60 cm × 90 cm	12 weeks
Chen and Zhong (2014)	Kunming mice; Female and Male; 3 weeks old	10	Running Wheels; Tunnels; Plastic colored toys; Shelters; Balls;	100 cm × 50 cm × 45 cm	6 weeks
Fernández et al. (2004)	Sprague–Dawley rats; Female and Male; 20 months old	10	Voluntary running; Tunnels; Toys;	0.8 m^2^	8 weeks
Herring et al. (2010)	TgCRND8 mice; Female; 5 months old	9	Tunnels; Balls; Soft materials; Varied locomotive substrates;	Not described	20 weeks
Jain et al. (2012)	Sprague–Dawley rats; Male; 3 months old	19	Plastic running wheel; Nesting material and an assortment of differently colored and texture plastic toys (balls, tubes, boxes, and bells).	35 × 9 × 20 in; 9 × 25 in. with two platform (20 × 9 × 15 in)	7 days
Prado Lima et al. (2018)	Wistar rats; Male; 3 weeks old	20	Running wheels; Toys; Balls; Ropes	50 cm × 50 cm × 50 cm	8 weeks
Mármol et al. (2015)	Long-Evans rats; Female and Male; 22 days old	8–16	Toys; Plastic balls; Tubes; Houses; Running wheels	45 cm × 35 cm × 50 cm	8 weeks
Mármol et al. (2017)	Long-Evans rats; Male; 3 weeks old	6	Running Wheel; Toys; and different objects	45 cm × 30 cm × 50 cm	8 weeks
Molina et al. (2021)	Wistar rats; Female and Male; 3 weeks old	3–5	Running Wheels; Tunnels; Ramps; Plastic toys;	40 cm × 25 cm × 16 cm	1–2 weeks
Montes et al. (2019)	Swiss-Webster mice, Male; 35–40 days age	5	Toys and Tunnels; 5 objects of different shapes, sizes, and textures	34 cm × 44 cm × 20 cm	4 weeks
Muhammad et al., 2017	Swiss Albino mice; Female, and Male; 4–5 weeks old	10	Tubes, ramps; stairs, and different toys (hard plastic balls, cubes, cones, and sticks)	66 cm × 46 cm wide × 38 cm	28 days
Pereira et al. (2009)	Wistar rats; Male; 7th postnatal day	7–10	Three floors; Ramps; Running Wheel and Several Objects with different shapes and textures	40 cm × 60 cm × 90 cm	9 weeks
Tapias et al. (2022)	Sprague Dawley rats; Male; 3-month-old	10–12	Toys (e.g., blocks, tubes, balls), nesting materials (e.g., bedding), and *ad libitum* food and water	92 cm × 78 cm × 51 cm	3 weeks
Thamizhoviya and Vanisree (2021)	Wistar Rats; Male; age group were not informed	6	Colorful rearrangeable tunnels; pipes; toys; diverse shapes; running wheel.	120 cm × 75 cm × 75 cm	28 days
Zhang et al. (2016)	Sprague Dawley rats; Male, Postnatal 21 day, and P34.	6	Running Wheel; Environmental complexity for social interaction and environmental novelty.	65 cm × 50 cm × 40 cm	14 days
Zhang et al. (2021)	Sprague Dawley rats; Male; 10 weeks old	12	Had climbing ladder; Chain; Tube of different shapes; Plastic tunnel; and Screen cover with color block and running wheel.	90 cm long × 75 cm wide × 50 cm high	28 days

Cm, Centimeters; m, meters; in, inch; wks, weeks; G, Groups; and P, Postnatal.

### 3.4 Environmental enrichment on oxidative stress biomarkers in brain areas of rodents exposed to normal and unfavorable environmental conditions

**Figure 2:**
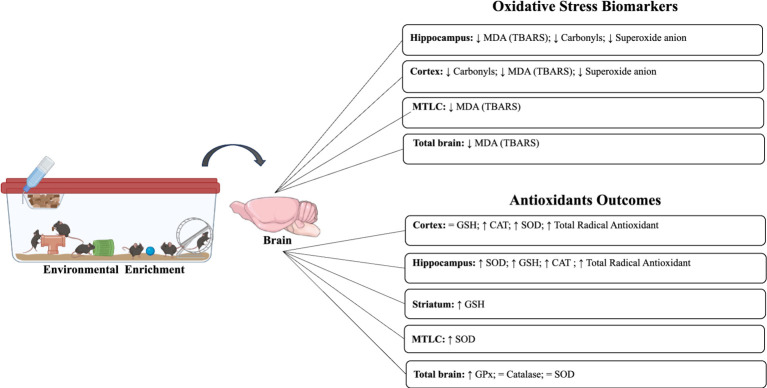
Impact of environmental enrichment on oxidative stress and antioxidant outcomes in experimental models subjected to normal environmental conditions.

**Figure 3:**
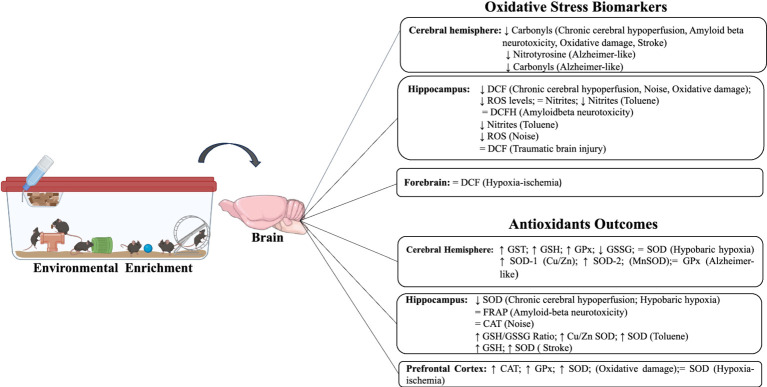
Impact of environmental enrichment on oxidative stress and antioxidant outcomes in experimental models subjected to unfavorable environmental conditions.

In the included studies, different biomarkers of OS were observed, such as carbonyls, DCF, MDA/TBARS, nitrotyrosine, ROS levels, 4-HNE, and superoxide anion. In the absence of unfavorable external environmental stimuli in the hippocampus, two studies evaluated carbonyl levels, which were significantly reduced after intervention with EE (Mármol et al., 2015, 2017). Furthermore, a reduction in MDA (TBARS) (*n* = 4) and superoxide anion levels was observed in the hippocampus, MTLC, cortex, and total brain (Cheng et al., 2014; Mármol et al., 2015, 2017; Muhammad et al., 2017; Figures 2, 3).

Under adverse conditions, similar results on MDA and carbonyl levels were also observed in the hippocampus, cerebral hemisphere, and forebrain when exposed to chronic cerebral hypoperfusion, Alzheimer-like model, hypobaric hypoxia, oxidative damage, and post-stroke (Herring et al., 2010; Cechetti et al., 2012; Jain et al., 2012; Prado Lima et al., 2018; Thamizhoviya and Vanisree, 2021; Zhang et al., 2021). Five studies evaluated DCF levels, three studies only in the hippocampus (Cechetti et al., 2012; Prado Lima et al., 2018; Molina et al., 2021), one study used the hippocampus and frontal cortex (Pereira et al., 2009), and another analyzed the forebrain only (Thamizhoviya and Vanisree, 2021). In the hippocampus, three studies observed a decrease in DCF after exposure to EE associated with chronic cerebral hypoperfusion, noise, and oxidative damage (Cechetti et al., 2012; Molina et al., 2021; Thamizhoviya and Vanisree, 2021). Two studies showed no significance in DCF levels after EE (Prado Lima et al., 2018; Tapias et al., 2022) (Table 4).

**Table 4:** Impacts of environmental enrichment on oxidative stress and antioxidant outcomes in experimental models subjected to normal environmental conditions.

Author, year	Tissue	Oxidative stress biomarkers	Antioxidants outcomes
Cheng et al. (2014)	Hippocampus; MTLC	↓ MDA (TBARS)	↑ SOD
Fernández et al. (2004)	Hippocampus; Total Cortex; Striatum	-	↑ GSH (Hippocampus and Striatum); ↔ GSH (Total Cortex)
Mármol et al. (2015)	Hippocampus; Cortex	↓ Carbonyls; ↓ MDA (TBARS); ↓ Superoxide anion	↑ CAT; ↑ SOD; ↑ Total Radical Antioxidant
Mármol et al. (2017)	Hippocampus	↓ Carbonyls; ↓ MDA (TBARS); ↓ Superoxide anion	↑ CAT; ↑ Total Radical Antioxidant
Muhammad et al. (2017)	Total brain	↓ MDA (TBARS)	↑ GPx; ↔ Catalase; = SOD

Cu/Zn SOD, Copper/zinc superoxide dismutase; CAT, Catalase; DCF, 2′,7′-Dichlorofluorescein; FRAP, Ferric reducing antioxidant power; GST, Glutathione S-transferase, GPx, Glutathione peroxidase; GSH, Reduced glutathione; GSSG, Oxidized glutathione; GSH/GSSG ratio, Reduced glutathione/oxidized glutathione; MDA, Malondialdehyde; MTLC, Medial-temporal lobe cortex; ROS levels, Reactive oxygen species; SOD, Superoxide dismutase; SOD-1, Superoxide dismutase-1; SOD-2, Superoxide dismutase-2. ↔: No significant difference (*p* > 0.05). ↓ Significant decrease; ↑ Significant increase.

Two studies evaluated ROS levels, one of them only in the hippocampus (Jain et al., 2012) and the other in the hippocampus and prefrontal cortex, after hypobaric hypoxia and oxidative damage, respectively (Montes et al., 2019). Both studies observed that EE was able to significantly reduce ROS levels. Two studies assessed 4-HNE levels in the hippocampus and prefrontal cortex (Zhang et al., 2016; Tapias et al., 2022). A significant decrease in 4-HNE levels after EE, traumatic brain injury, and hypoxia was observed. Finally, two studies evaluated other compounds related to OS. Nitrotyrosine (Herring et al., 2010) was evaluated in the cerebral hemisphere, and nitrites in the hippocampus and prefrontal (Montes et al., 2019), both of whom observed that EE was able to decrease their levels after the Alzheimer-like model and toluene exposure (Table 5).

**Table 5:** Impacts of environmental enrichment on oxidative stress and antioxidant outcomes in experimental models subjected to unfavorable environmental conditions.

Author, year	Tissue	Unfavorable environmental condition	Oxidative stress biomarkers	Antioxidants outcomes
Cechetti et al. (2012)	Hippocampus	Chronic cerebral hypoperfusion	↓ DCF; ↓ MDA (TBARS)	↓ SOD
Herring et al. (2010)	Cerebral hemisphere	Alzheimer-like	↓ Carbonyls; ↓ Nitrotyrosine	↑SOD-1 (Cu/Zn SOD); ↑SOD-2 (MnSOD); ↔ GPx
Jain et al. (2012)	Hippocampus	Hypobaric hypoxia	↓ MDA (TBARS); ↓ ROS levels	↑ GST; ↑ GSH; ↑ GPx; ↓ GSSG; ↔ SOD
Prado Lima et al. (2018)	Hippocampus	Amyloid beta neurotoxicity	= DCFH; ↓ MDA (TBARS)	↔ FRAP
Molina et al. (2021)	Hippocampus	Noise	↓ DCF ↓ ROS	↔ CAT
Montes et al. (2019)	Hippocampus; Prefrontal Cortex	Toluene	↓ Nitrites; ↓ ROS levels (Hippocampus) = Nitrites; ↓ ROS levels (Prefrontal Cortex)	↑ GSH/GSSG Ratio; ↑ Cu/Zn SOD; ↑SOD
Pereira et al. (2009)	Hippocampus; Prefrontal Cortex	Hypoxia-ischemia	= DCF	↔ SOD
Tapias et al. (2022)	Hippocampus	Traumatic brain injury	Ipsilateral: ↓ 4 HNE Contralateral: = DCF	-
Thamizhoviya and Vanisree (2021)	Forebrain	Oxidative damage	↓ DCF; ↓ MDA (TBARS); ↓ ROS levels	↑ CAT; ↑ GPx; ↑ SOD
Zhang et al. (2016)	Prefrontal cortex	Hypoxia	↓ 4 HNE	-
Zhang et al. (2021)	Hippocampus	Stroke	↓ MDA (TBARS)	↑ GSH; ↑ SOD

Cu/Zn SOD, Copper/zinc superoxide dismutase; CAT, Catalase; DCF: 2′,7′-Dichlorofluorescein; FRAP, Ferric reducing antioxidant power; GST, Glutathione S-transferase; GPx, Glutathione peroxidase; GSH, Reduced glutathione; GSSG, Oxidized glutathione; GSH/GSSG, Ratio reduced glutathione/oxidized glutathione; MDA, Malondialdehyde; MTLC, Medial-temporal lobe cortex; ROS levels, Reactive oxygen species; SOD, Superoxide dismutase; SOD-1, Superoxide dismutase-1; SOD-2, Superoxide dismutase-2. ↔: No significant difference (*p* > 0.05).↓ Significant decrease; ↑ Significant increase.

### 3.5 Environmental enrichment and antioxidant response in brain areas of rodents exposed to normal and unfavorable environmental conditions

In 14 included studies, it was observed that there was variability in markers responsible for mediating the antioxidant response, such as catalase, FRAP, GPx (GSH-px), GST, GSH, GSSG, GSH/GSSG ratio, SOD, SOD-1 (Cu/Zn SOD), SOD-2 (MnSOD), and total radical antioxidant. In the absence of environmental damage, two studies observed an increase in catalase enzyme activity in the hippocampus and cortex after EE but not in the total brain (Mármol et al., 2015, 2017). Increases in total antioxidant radical activity were also observed in the same areas (Mármol et al., 2015, 2017). SOD levels were elevated in two studies (hippocampus, MTLC, and cortex; Cheng et al., 2014; Mármol et al., 2015), whereas, in the whole brain, no differences were observed after EE (Muhammad et al., 2017). Similarly, increases in GSH and GPx levels were observed in the hippocampus, striatum, and total brain (Fernández et al., 2004; Muhammad et al., 2017). Only in the total cortex, no differences were observed in GSH (Fernández et al., 2004) (Table 5).

Under adverse conditions, two studies assessed the impact of EE on the activity of catalase levels after exposure to noise and oxidative damage. In the hippocampus, no difference was observed in catalase activity (Molina et al., 2021), whereas in the forebrain, an increase was observed after EE (Thamizhoviya and Vanisree, 2021). Only one included study evaluated FRAP levels and found no significant difference in the hippocampus after amyloid beta neurotoxicity associated with EE (Prado Lima et al., 2018).

Three included studies evaluated GPx activity after an Alzheimer-like model, hypobaric hypoxia, and oxidative damage. One study evaluated the cerebral hemisphere and found no significant differences after EE (Herring et al., 2010). However, in the forebrain, an increase in GPx activity was observed after EE (Thamizhoviya and Vanisree, 2021). Similarly, in the hippocampus, a significant increase in GPx activity was also observed after intervention with EE (Jain et al., 2012). GST activity was evaluated in only one included study, and it demonstrated a significant increase in the hippocampus after hypobaric hypoxia and EE (Jain et al., 2012).

Two included studies assessed GSH levels in conditions of hypobaric hypoxia and stroke. These studies observed a significant increase in GSH levels only in the hippocampus after EE (Jain et al., 2012; Zhang et al., 2021). Only one study, in the hippocampus evaluated the levels of GSSG, in which it identified a significant decrease after exposure to EE and hypobaric hypoxia (Jain et al., 2012). One included study evaluated the GSH/GSSG ratio in two brain areas, the hippocampus and prefrontal cortex. After EE, there was an increase in both tissues after toluene exposure (Montes et al., 2019).

Seven included studies evaluated SOD activity after unfavorable environmental exposure. Three studies evaluated SOD in the hippocampus only, in which there was heterogeneity of responses produced by EE. One of the studies showed a significant increase (Zhang et al., 2021), another a significant decrease (Cechetti et al., 2012), and one included study did not observe a significant difference (Jain et al., 2012). In these studies, different environmental conditions were observed (chronic cerebral hypoperfusion, hypoxia-ischemia, and stroke). One study evaluated SOD-1 (Cu/ZnSOD) and SOD-2 (MnSOD) activities in the cerebral hemisphere, and the authors observed a significant increase after EE and Alzheimer-like models (Herring et al., 2010). In the forebrain, an increase in SOD activity was identified after the intervention with EE and oxidative damage (Thamizhoviya and Vanisree, 2021). Another included study observed the same response in the hippocampus and prefrontal cortex on SOD activity (Montes et al., 2019). To see summarized resumes, check Figures 2, 3.

## 4 Discussion

Environmental enrichment is known as an experimental approach for brain improvement based on social stimulation via sensory, motor, social, and/or cognitive nested mechanisms (Kempermann, 2019). Relying on the molecular, physiological, and social aspects, EE affects many domains of brain function by modulating from gene expression to global phenotypes. Thus, in this systematic review, we investigated the brain oxidative balance, as one of the molecular outcomes of EE in rodents exposed or not to brain-related impairments.

Since the EE paradigm arose, it has been described as having effects on behavior, especially learning and memory capacity (Balietti and Conti, 2022). Notably, synaptic plasticity-related memory drives our attention to the hippocampus, which represents the major structure evaluated in the studies selected here. Lying in the medial temporal lobe of the brain, the hippocampus acts actively in mammal neurogenesis, wherein the oxidative balance fluctuates throughout life, especially within the differentiation of neural and/or astroglia lineage; thus, the ability to deal with ROS-related transient stress is crucial to the central nervous system (CNS) health (Huang et al., 2015).

In healthy animals, EE, regardless of type and duration, downregulates OS biomarkers in the CNS, mainly in the hippocampus (Cheng et al., 2014). It is critical for growing animals, that these control OS, especially where the brain developmental process is still prominent and requires a tuning environment for neural development (Morgane et al., 2002). Noteworthy, CAT was the major antioxidant enzyme upregulated in healthy animals exposed to EE, providing a greater ability to deal with H2O2, which, due to its molecular properties, has an increased membrane permeability and can act as a neuromodulator in pathways with different lifetimes (Rice, 2011).

Although EE promoted a neuroprotector effect by reducing oxidative damage, only two studies evaluated both ROS production and removal, we are unable to determine what/how compounds from each arm of the oxidative balance were modulated by EE. Besides, it seems that as the animals get older, their antioxidant enzymes become less responsive to EE protocols, reinforcing the importance of diet-related antioxidant compounds. Compelled by the effects of EE on the CNS of healthy animals, our review further discusses the application of EE as a tool against harmful insults in the CNS. In adverse conditions, EE also demonstrated a positive effect on the oxidative balance. The studies summarized here suggest an overall increase in the antioxidant capacity, both enzymatic and non-enzymatic, which are the main factors for the neuroprotective effects in the CNS under unfavorable conditions.

The endogenous antioxidant enzymes, such as cytosolic and mitochondrial superoxide dismutase as well as glutathione peroxidase, might decelerate oxidative damage-associated neural dysfunction, especially in the prefrontal structure. It is important to point out that, among the harmful conditions included in this review, only in acute stress (immobilization) was EE able to ameliorate all oxidative parameters evaluated, including the enzymes cited above. We believe that this phenomenon correlates with the hormesis effect, as an acute stressful event transiently increases ROS production, triggering a compensatory response in the antioxidant defense, as largely described in exercise training protocols (Ji et al., 2006; Radak et al., 2008).

Furthermore, like SOD, other antioxidant compounds might be differently distributed across the CNS, which may explain why similar EE protocols have been followed by converse outcomes. In any case, it is important to highlight that the augmented dismutation of superoxide anion led by EE represents a stronger antioxidant network, crucial in the encounter of several hypoxic conditions and some neurodegenerative disorders (Lindenau et al., 2000).

This is the first systematic review that addresses the impacts of EE protocols on cerebral oxidative balance in rodents exposed to favorable and unfavorable environmental conditions, including models of chronic cerebral hypoperfusion, Alzheimer’s disease, hypobaric hypoxia, amyloid beta neurotoxicity, ischemia-hypoxia, brain damage due to traumatic situations, hypoxia, stroke, oxidative damage, and exposure to toluene. The proposal to address these different exposure conditions to environmental conditions demonstrates the effectiveness of EE in significantly reducing markers linked to the production of OS, such as superoxide anion, DCF, MDA, carbonyls, 4-HNE, and ROS levels in brain regions important for the functioning of the body. Furthermore, its ability to significantly increase components of enzymatic (SOD, CAT, and GST), as well as non-enzymatic (GPx, GSH, GSSG, and REDOX state [GSH/GSSG ratio]) antioxidant defenses.

Among the studies selected here, just three explore the possible mechanisms involved in the EE-related oxidative balance improvements, wherein both pro-oxidant and antioxidant compounds have been modulated. Zhang et al. (2016) proposes that the neuroprotective effects of EE against oxidative damage rely on NADPH oxidase-related ROS reduction, wherein its reduced expression and activity downregulate the overall ROS production (Bedard and Krause, 2007). Along with the pro-oxidant reduction, EE boosts antioxidant defenses by upregulating the nuclear factor erythroid 2-related factor 2 (Nrf2) pathway (Zhang et al., 2021), which modulates GSH levels as well as the expression of SOD, Heme oxygenase 1 (HO1), and NADP(H) quinone oxidoreductase 1 (NQO1), a FAD-dependent protein with cytoprotective and antioxidant functions (Dinkova-Kostova and Talalay, 2010). Additionally, the decrease in OS can modulate itself through the mitogen-activated protein kinase (MAPK) family, diminishing the transduction of stress-activated protein kinases (SAPK)/Jun amino-terminal kinases (JNK), which reduces inflammatory signals, such as prostaglandin E2 receptor (Herring et al., 2010; Davies and Tournier, 2012).

In summary, the direct or indirect modulation of the oxidative balance contributes to protection against cellular oxidative damage, which is related to the pathophysiology of several chronic degenerative diseases, including different types of cancer, cardiovascular diseases, and especially neurodegenerative diseases. In this sense, the use of non-pharmacological tools, such as the EE approach, emerges as a viable and low-cost alternative for preventive containment of these damages, and their application may be considered translationally in studies with humans. Finally, the structure of each EE protocol must be considered in terms of its structure, size of the space (centimeters, millimeters, and meters, height, length, and width), duration in weeks or months, quantity and types of objectives (plastics and/or wood), and cleaning conditions to guarantee a greater standardization capacity, thus being able to better understand its effects.

## 5 Limitations and strengths

Although the EE paradigm has been extensively described, the variability of set-ups makes direct comparisons among the studies difficult, limiting our further discussion. In addition, the “clutter” cages make tracking animals throughout the objects, as in physical exercise protocols, tough. Still, regardless of those changing settings, the overall positive outcomes, along with the non-invasive and relatively simple procedure, are advantages of the EE approach.

In any case, studies have suggested basic parameters that must be included in any EE protocol, such as: (I) bigger cage size; (II) increased social interaction; (III) hide-out boxes; (IV) climbing objects; (V) toys that provide somatosensory stimulus in different categories; (VI) augmented physical activity; and (VII) changes in the EE layout. Detailed information can be found elsewhere (Ismail et al., 2021; Love et al., 2022).

## 6 Conclusion

In conclusion, our systematic review demonstrated that EE is a valid tool for the improvement of the oxidative balance in the CNS, wherein the hippocampus has been the main structure studied and affected. The overall decrease in OS biomarkers indicates a refinement in ROS detoxification, which is differently modulated by the health status of the rodents. Healthy animals have a higher capacity to deal with peroxides, while injured animals reinforce their superoxide detoxification, triggering an improvement in the antioxidant network. From the extensive analysis conducted in our systematic review, it is evident that EE serves as a valuable intervention for enhancing oxidative balance within the CNS, with a predominant focus on the hippocampus. This comprehensive scrutiny revealed a noteworthy reduction in biomarkers associated with OS across various brain areas. Notably, the efficacy of EE varied based on the health status of the rodents, displaying a dual effect: augmenting peroxide management in healthy subjects and bolstering the detoxification of superoxide in injured animals. This modulation ultimately contributes to an enhanced antioxidant network, showcasing the nuanced and adaptive nature of EE’s impact on oxidative balance within the CNS.

## Data availability statement

The original contributions presented in the study are included in the article/supplementary material, further inquiries can be directed to the corresponding authors.

## Author contributions

TR: Conceptualization, Data curation, Investigation, Methodology, Writing – original draft, Writing – review & editing. MS: Conceptualization, Data curation, Investigation, Methodology, Writing – original draft, Writing – review & editing. DS: Data curation, Methodology, Writing – original draft, Writing – review & editing. GJ: Data curation, Methodology, Writing – original draft, Writing – review & editing. RBA: Conceptualization, Data curation, Methodology, Writing – original draft, Writing – review & editing. DF: Data curation, Investigation, Methodology, Writing – original draft, Writing – review & editing. RS: Data curation, Methodology, Writing – original draft, Writing – review & editing. GB: Methodology, Project administration, Resources, Supervision, Writing – original draft, Writing – review & editing. FY: Methodology, Project administration, Resources, Supervision, Writing – original draft, Writing – review & editing, Investigation. BY: Methodology, Resources, Supervision, Writing – original draft, Writing – review & editing. RMA: Methodology, Resources, Supervision, Writing – original draft, Writing – review & editing. FS: Data curation, Investigation, Methodology, Writing – original draft, Writing – review & editing. CL: Conceptualization, Investigation, Methodology, Project administration, Supervision, Writing – original draft, Writing – review & editing.
